# Genome-wide analysis validates aberrant methylation in fragile X syndrome is specific to the *FMR1* locus

**DOI:** 10.1186/1471-2350-14-18

**Published:** 2013-01-29

**Authors:** Reid S Alisch, Tao Wang, Pankaj Chopra, Jeannie Visootsak, Karen N Conneely, Stephen T Warren

**Affiliations:** 1 Genetics and Molecular Biology Graduate Program, Emory University, Atlanta, GA, 30322, USA; 2 Departments of Human Genetics, Emory University School of Medicine, 615 Michael Street, Atlanta, GA, 30322, USA; 3 Departments of Biochemistry, Emory University School of Medicine, 615 Michael Street, Atlanta, GA, 30322, USA; 4 Departments of Pediatrics, Emory University School of Medicine, 615 Michael Street, Atlanta, GA, 30322, USA; 5 Current address: Department of Psychiatry, University of Wisconsin–Madison, Madison, WI, 53719, USA

**Keywords:** Epigenetics, DNA methylation, Fragile X syndrome

## Abstract

**Background:**

Fragile X syndrome (FXS) is a common form of inherited intellectual disability caused by an expansion of CGG repeats located in the 5^′^ untranslated region (UTR) of the *FMR1* gene, which leads to hypermethylation and silencing of this locus. Although a dramatic increase in DNA methylation of the *FMR1* full mutation allele is well documented, the extent to which these changes affect DNA methylation throughout the rest of the genome has gone unexplored.

**Methods:**

Here we examined genome-wide methylation in both peripheral blood (N = 62) and induced pluripotent stem cells (iPSCs; N = 10) from FXS individuals and controls.

**Results:**

We not only found the expected significant DNA methylation differences in the *FMR1* promoter and 5^′^ UTR, we also saw that these changes inverse in the *FMR1* gene body. Importantly, we found no other differentially methylated loci throughout the remainder of the genome, indicating the aberrant methylation of *FMR1* in FXS is locus-specific.

**Conclusions:**

This study provides a comprehensive methylation profile of FXS and helps refine our understanding of the mechanisms behind *FMR1* silencing.

## Background

Individuals with fragile X syndrome (FXS) exhibit a broad range of phenotypes, including varying degrees of intellectual disability, social impairment, macroorchidism, and an elongated face with large, everted ears. The most common mutation that causes FXS is an expansion of the CGG trinucleotide repeat located within the 5^′^ untranslated region (UTR) of the *FMR1* gene [1-3]. When this expansion is greater than 200 repeats (known as the full mutation), the *FMR1* promoter becomes hypermethylated, which prevents the expression of *FMR1*. Deletions and sequence variants within *FMR1* result in a very small fraction of FXS cases [4-6], arguing that the loss of *FMR1* function is the cause of FXS.

An association between the hypermethylation of the *FMR1* trinucleotide repeats and FXS was first recognized over two decades ago [7], which sparked intensive study of the DNA methylation dynamics within the *FMR1* locus. Research into the developmental timing of *FMR1* silencing in chorionic villi (CV) samples from FXS patients has revealed that the FXS full mutation alleles are still expressed during early embryogenesis (i.e. during gastrulation), indicating that epigenetic repression is established at a later developmental time point [8]. Furthermore, human embryonic stem cells (hESCs) derived from FXS patient embryos also express *FMR1* in undifferentiated cells, until cellular differentiation triggers the recruitment of specific histone modifications, followed by DNA methylation and subsequent silencing of *FMR1* transcription [9]. In contrast, the *FMR1* locus remains hypermethylated in induced pluripotent stem (iPS) cell lines derived from FXS patients, suggesting that, once the methylation marks are established at this locus, they are stable and resistant to current reprograming methodologies [10].

A long-standing question in the field is whether the full mutation triggers DNA methylation elsewhere in the genome or only at the *FMR1* locus. Resolving this question could modify theories of how an expanded CGG repeat triggers aberrant DNA hypermethylation. For example, RNA-induced transcriptional silencing (RITS) has been proposed as a mechanism to explain the silencing of *FMR1*[11]. RITS is a form of gene silencing triggered by small interfering RNA (siRNA) and generally causes the transcriptional downregulation of a genomic region [12]. This model is attractive in that the unmethylated full mutation allele is known to be expressed in early development, presumably producing a transcript with a long riboCGG tract, and this riboCGG tract is cleaved *in vitro* by Dicer[13], producing small siRNA-like fragments of the riboCGG tract. Thus, it may be that small CGG RNAs could target chromatin-modifying activities back to the *FMR1* locus. If true, there could be other CGG tracts in the genome that also are modified by this mechanism. To test this hypothesis, we examined DNA methylation levels at nearly half a million sites throughout the genome in the peripheral blood and fibroblast iPS cells of FXS patients using a highly sensitive genome-wide assay that quantitates methylation level at single CpG dinucleotide resolution; our results show that the hypermethylation of the *FMR1* locus in FXS is indeed locus-specific.

## Methods

### Human samples

The study protocol and consent form used in this investigation wasreviewed and approved by the Emory Internal Review Board on August 3, 2012and given the approval number CR8_IRB00001764.

### Derivation of iPS cells

Human normal fibroblasts CRL2097 were obtained from ATCC, and GM0011 (normal), GM05848, and GM07730 (fragile X patients) were obtained from the Coriell Cell Repositories. The fibroblasts were cultured in DMEM containing 10% FBS, 1× glutamine, 1× Non-Essential amino acids, and 1× Pen/Strep.

For human iPSC reprogramming, 1 × 10^5^ fibroblasts were seeded in a well of a 6-well plate. The next day, concentrated pMXs-hOCT4, hSOX2, hKLF4, and c-hMYC retrovirus were added to cells in the presence of 6 μg/ml polybrene. A second round of transduction was repeated the following day. On day 7 after initial transduction, the cells were reseeded in 10-cm dishes with irradiated MEF feeders. The hiPSC colonies were picked between days 18–25. iPSCs were maintained in hiPSC standard medium (DMEM/F12, 20% KnockOut Serum Replacement, 1× MEM Non-Essential Amino Acids, 1× glutamine, 0.11 mM 2-mercaptoethanol, 10 ng/ml bFGF) on irradiated MEF feeders. The established iPSC cell lines were subsequently confirmed with AP staining, and pluripotent markers by immunofluorescence staining and the ability to differentiate into 3 germ layers. Before isolating genomic DNA, iPS cells were subcultured in mTeSR1 feeder free system (STEMCELL Technologies) for at least 3 passages to reduce potential contamination.

### DNA methylation profiling

Five hundred nanograms of human genomic DNA was sodium bisulfite–treated for cytosine (C) to thymine (T) conversion using the EZ DNA Methylation-Gold kit (Zymo Research). The converted DNA was purified and prepped for analysis on the Illumina HumanMethylation450 BeadChips following the manufacturer’s guidelines. Briefly, converted DNA was amplified, fragmented, and hybridized to the HumanMethylation450 pool of allele-differentiating oligonucleotides. After a series of extension, ligation, and cleanup reactions, the DNA was labeled with a fluorescent dye. The labeled DNA was then scanned using an Illumina BeadArray Reader or iScan. Image analysis and signal determination were performed using the GenomeStudio software, Methylation Module (Illumina).

### Interpretation and QC of DNA methylation data

CpG DNA methylation data were interpreted using GenomeStudio to quantify methylated (M) and unmethylated (U) signal intensities for genomic DNA. The signals were quantile normalized separately, and overall methylation levels (β) were calculated as the ratio of methylated to total signal [i.e. β = M/(M + U + 100)], where β ranges from 0 (unmethylated) to 1 (methylated). Quality control of data resulted in removal of samples with aberrantly low signal intensity (mean <2000) or with fewer than 90% of CpG loci detected, where a given locus was deemed not detected if the detection P-value was >0.01 (detection P-value provided by GenomeStudio and calculated relative to background signal). Any probe having more than 25% detected P-values >0.01 was discarded from the analysis. Missing data were imputed using the “impute.knn” function from the “impute” package in R (Cran). Assay controls were inspected to remove samples with poor bisulfite conversion, staining, extension (single nucleotide extension assay), hybridization, or specificity. Furthermore, outliers identified by hierarchical clustering and/or dissimilarity matrices were removed. Additionally, one control DNA replicate was run on each BeadChip to assess overall assay reproducibility. Methylation profiles of the control DNA correlated well, with an average Pearson correlation coefficient (R) of 0.990 between replicates.

### Analysis of FXS-associated CpG loci

To analyze DNA methylation differences associated with FXS, we fit a separate regression for each CpG site. Although samples were randomly distributed across BeadChips and experiments with respect to disease, BeadChip was also included as a random effect covariate in all analyses to account for potential batch effects. The package “nlme” in R (Cran) was used for the mixed effect model. Fixed effects included in the model were intensity, position, and age (for blood). To test several *FMR1*-related and -unrelated hypotheses, we filtered the data to include only those probes that reside in the following genomic regions: 1500 base pairs of a transcription start site; 200 base pairs of a transcription start site; the first exon of a gene; the “3^′^UTR” of a gene; the “5^′^UTR” of a gene; a CpG Island; the “N_Shore” or “S_Shore” of a CpG island; or near a CGG trinucleotide repeat containing at least 8 consecutive repeats. We also filtered the data to include only those probes annotated to genes found to play a role in recurrent genomic abnormalities. To correct for multiple hypothesis testing, we applied a Benjamini-Hochberg False Discovery Rate (FDR) correction using the R function “p.adjust,” but to avoid false positives due to the small sample size, we used conservative Bonferroni adjustment for our ultimate determination of significance.

### Permutation analyses

All permutation analyses were conducted in R using the same linear model as the actual analysis, where BeadChip was treated as a mixed-effects covariate, but in each permutation the disease status of the subjects was randomly reassigned. In total, 1000 permutations were conducted for both the peripheral blood and iPS cell groups independently. Permutation P-values for each CpG locus were calculated by assessing the number of times each locus was more significantly associated with FXS in the 1000 permuted data sets than the actual association (Additional file 1: Figure S3; Additional file 2: Table S1 and Additional file 3: Table S2).

### Data access

We have submitted the data generated from the 9 FXS samples and the 53 controls for this study to the Gene Expression Omnibus (GEO), which can be found under the Gene Series: GSE41273.

## Results

**Figure 1:**
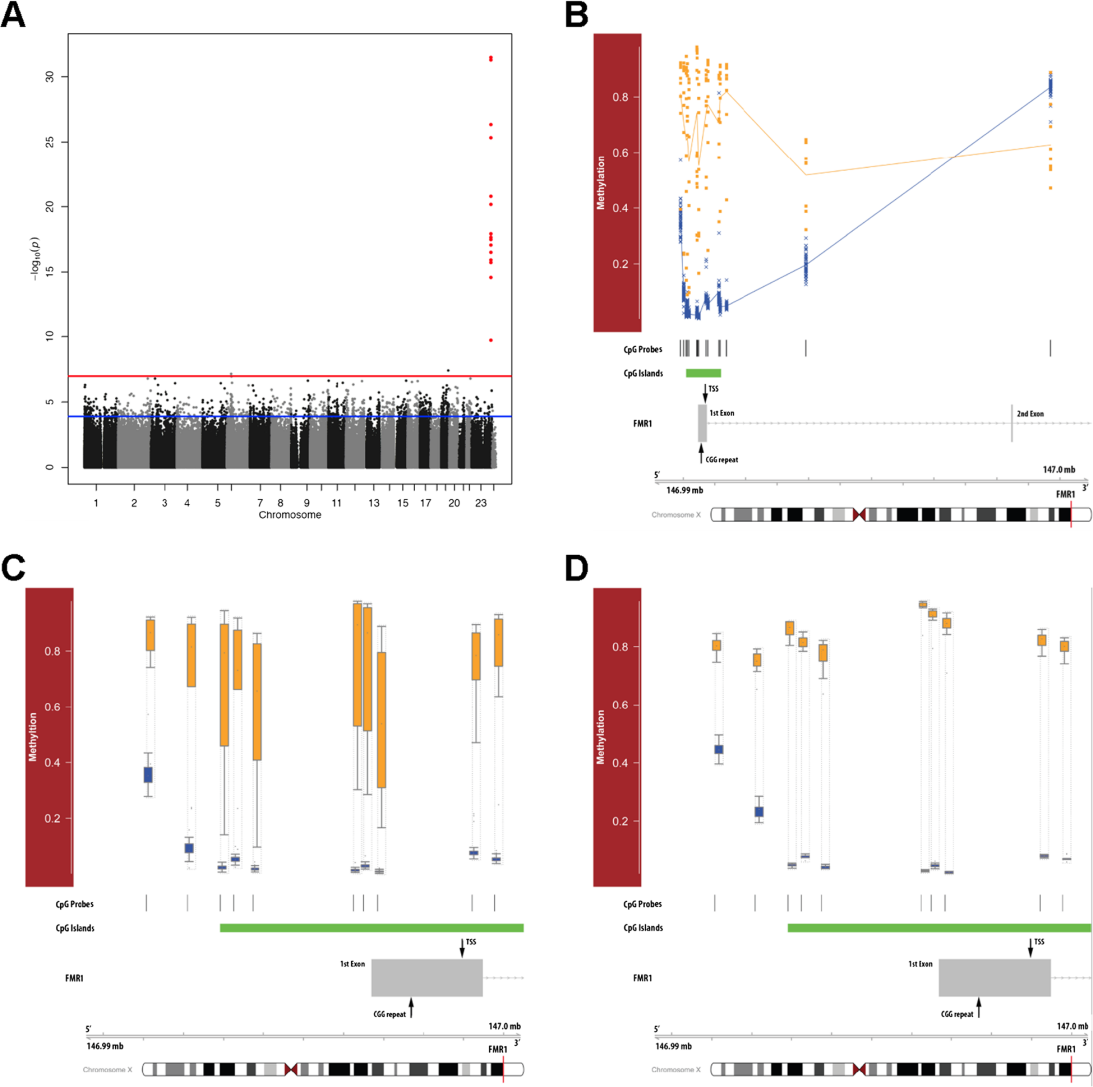
**FXS-associated differentially methylated loci.(A)** Modified Manhattan plot of FXS-associated methylation levels in the peripheral blood: loci are displayed with the –log10(*P*-value) generated by the linear mixed-effect model (y-axis). Horizontal lines reflect cutoffs for FDR <0.05 (blue line) and Bonferroni-adjusted P-value <0.05 (red line). *FMR1* annotated loci are shown in red; otherwise, loci are colored black or gray on alternating chromosomes (x-axis). **(B)** Relative locations of *FMR1* methylation. Top panel shows the methylation levels (y-axis) in FXS (orange squares) and control (blue crosses) individuals at several loci annotated in or near the *FMR1* locus. Shown below are the relative CpG coverage (vertical black lines) and CpG island location (green rectangle). Bottom panel depicts a gene schematic of *FMR1*, indicating the relative location of the CGG repeat (expanded in FXS), the *FMR1* transcription start site (TSS), and exons 1 and 2. The ideogram of the X chromosome (bottom) shows the relative location of *FMR1* (red vertical bar), and the genomic position shown above is relative to HG-19 coordinates. **(C and D)***FMR1* promoter DNA methylation levels in peripheral blood and iPS cells. Top panel shows box and whisker plots of the methylation levels (y-axis) in FXS (orange) and control (blue) individuals at several loci annotated in the *FMR1* promoter. Bottom panel is described in **B**.

We investigated FXS-associated ectopic DNA methylation changes using DNA extracted from the peripheral blood of nine FXS individuals and 53 healthy males. At the time of collection, these individuals ranged in age from >1–48 years (median 7.8; FXS; Additional file 4: Figure S1) and 3–18 years (median 10.3; controls; Additional file 4: Figure S1) and were epityped using Infinium HumanMethylation450 BeadChips, which provide a quantitative measure of DNA methylation denoted as β, calculated as the ratio of methylated to total DNA. This is a highly reproducible and widely used assay ([14-18] that measures β at 485,512 CpG dinucleotides located proximal to the promoters of nearly all RefSeq genes. To determine whether FXS-associated DNA methylation changes were present in these individuals, we analyzed each locus using a linear mixed-effects regression model that adjusted for age (see Methods) and identified 17 differentially methylated probes, 15 of which are annotated to the *FMR1* promoter or gene body: 14 FXS-methylated loci and 1 FXS-demethylated locus (Bonferroni <0.05; Figure 1 A, B; Additional file 2: Table S1 and Additional file 3: Table S2). Though the 15 *FMR1*-associated loci exhibit a unique FXS-associated DNA methylation profile of distinct methylation levels with little overlap for FXS samples versus controls (Figure 1B), the two autosomal differentially methylated loci (*KLK15* and *MICA*) were not so distinct; they were also hypomethylated rather than hypermethylated and were not flanked by other significantly differentially methylated loci, as one sees at *FMR1* (Additional file 5: Figure S2). This suggests these two autosomal loci are not modified similar to *FMR1* in full mutation individuals, and the results for these two loci may be false positives due to our small sample size or possible population substructure of the epigenome. To validate our methods statistically and adjust for possible failure of asymptotic assumptions due to the small sample size, we performed a thousand permutations of the data (see Methods). For the 15 *FMR1*-associated probes, the P-values from the original analysis were always lower than the 1000 P-values obtained in the permuted datasets, suggesting an empirical P-value of < .001 (Additional file 1: Figure S3; Additional file 2: Table S1; Methods). Since differential methylation of the *FMR1* allele is well established [7] and in direct agreement with our findings, these differences were not validated using an alternate molecular assay.

We next tested several FXS-related and -unrelated hypotheses, including recurrent genomic abnormalities associated with intellectual delay and annotated genomic structures (e.g. CpG islands; Methods). Although this approach reduces our multiple testing burden and effectively increases our power to find subtle yet significant changes in DNA methylation, as before the only probes identified as differentially methylated were probes annotated to the *FMR1* gene (data not shown). Note that even for the strict Bonferroni criteria employed for the genome-wide analysis, 75% of the CpG sites had >80% power to detect β – value differences of 0.10 or greater, so the lack of observed widespread methylation differences in genes other than *FMR1* cannot be explained by low power. Finally, to gain insight into the potential mechanism(s) behind FXS-associated DNA methylation changes, we annotated the genome for all CGG trinucleotide repeats containing at least eight consecutive repeats (N = 136 tracts; N = 452 probes; see Methods) and found two juxtaposed probes annotated to *ZFHX3* that reached significance (Additional file 6: Table S3). In contrast to the *FMR1* locus, the *ZFHX3* locus had no distinct FXS-associated DNA methylation or gene expression differences (Additional file 7: Figure S4; data not shown). These findings imply that the significant DNA methylation differences observed at the two probes do not have a functional consequence on *ZFHX3* expression. Together, these data suggest that the FXS-associated hypermethylation of the *FMR1* promoter is locus-specific and does not alter DNA methylation elsewhere in the genome.

To corroborate these findings, we derived a total of ten iPS cell lines from fibroblasts of two FXS patients (FXS-iPS) and two control individuals (iPS). DNA was extracted from 12 FXS-iPS cell lines and 11 iPS cell lines and epityped using Infinium HumanMethylation450 BeadChips. Limiting the FXS-associated DNA methylation analysis in this group to only those loci that satisfied an FDR <0.05 in the peripheral blood analysis (N = 1183 probes; Figure 1A; see Methods) yielded results similar to those found in peripheral blood: eight FXS-methylated loci and one FXS-demethylated locus; all nine differentially methylated loci are annotated to the *FMR1* promoter (Bonferroni <0.05) (Figure 1C; Additional file 8: Table S4). Since iPS cells show significant reprogramming variability, we also excluded the hotspots of aberrant reprogramming regions reported by Lister et al. [19] and still found FXS-associated DNA methylation changes only at the *FMR1* locus (data not shown). Subsequent hypothesis- and mechanism-driven analyses also failed to uncover any non-*FMR1* annotated FXS-associated DNA methylation changes, including at the *ZFHX3* locus. Therefore, FXS-derived iPS cells exhibit similar genome-wide methylation profiles as terminally differentiated cells of blood, a *FMR1*-specific epigenetic disruption.

## Discussion

This study provides a sensitive and comprehensive quantitative analysis of genome-wide DNA methylation levels in a group of FXS and control individuals. We found that FXS-associated hypermethylation is profound throughout the CpG island encompassing the *FMR1* 5^′^ UTR, revealing CpG dinucleotides whose distinct FXS methylation profile could improve current diagnostic methods. For example, there are four CpG loci in *FMR1* that show a clear distinction between all FXS and control samples with no overlap (Figure 1B), suggesting that interrogation of these loci for methylation would be diagnostic. The finding that FXS-associated methylation is significantly decreased at one CpG in the *FMR1* gene body is consistent with previous reports indicating that gene body hypermethylation is associated with active gene expression [20]. It would be interesting to explore whether this trend persists throughout the remainder of the gene.

Our examination reported here shows that CGG repeats elsewhere in the genome do not appear abnormally methylated in *trans* with the full mutation. Thus, either the hypothesis of a RITS role in silencing of *FMR1* is false, or there may be a threshold of length for a CGG repeat tract to be susceptible to silencing, since there are no known CGG tracts in the reference genome that even approach the size of a premutation, let alone a full mutation allele. Indeed, the expression of the normal *FMR1* allele opposite the full mutation in FXS females would be consistent with a threshold model.

## Conclusion

When *FMR1* was first identified, the question posed in this study was unanswerable. Today, our knowledge of the human genome sequence allows genome-wide examination of DNA methylation differences. Here we report that only probes located in the *FMR1* promoter or gene body exhibit FXS-associated DNA methylation differences in DNA from peripheral blood and iPS cells of FXS individuals. Thus, while this study does not determine the mechanism behind the aberrant methylation in the expanded *FMR1* repeat, it does help refine our mechanistic picture of *FMR1* silencing in fragile X syndrome. Since we did not find any non-*FMR1*-associated differentially methylated loci, we have made a significant stride toward finally put a long-standing question in FXS research to rest.

## Abbreviations

FXS: Fragile X syndrome; UTR: Untranslated region; CV: Chorionic villi; hESCs: Human embryonic stem cells; iPS: Induced pluripotent stem; C: Cytosine; T: Thymine; M: Methylated; U: Unmethylated; β: Methylation levels; R: Pearson correlation coefficient; RITS: RNA-induced transcriptional silencing; siRNA: Small interfering RNA.

## Competing interests

The authors declare that they have no competing interests.

## Authors’ contribution

RSA designed experiments, generated and interpreted all methylation data and wrote manuscript. TW designed experiments, generated expression data and wrote manuscript. PC designed and analyzed all methylation data. JV designed recruitment protocol and collected samples. KNC developed analytical methods of methylation data. STW designed experiments, interpreted data and wrote manuscript. All authors read and approved the final manuscript.

## Pre-publication history

The pre-publication history for this paper can be accessed here:

http://www.biomedcentral.com/1471-2350/14/18/prepub

## Supplementary Material

Additional file 1: Figure S3 Permutation analysis of FXS-associated loci. Scatterplot of permuted (1000 permutations) FXS-associated *P*-values (x-axis) compared to asymptotic *P*-values (y-axis) calculated using the linear model (Pearson R = 0.997). Click here for file

Additional file 2: Table S1 Summary of the linear mixed-effects regression model. Column headers include: Illumina identification name (cpgids); Bonferroni-corrected P-values (bonferroni); False discovery rate P-values (fdr); Uncorrected P-value (raw_pvalue); Permutation P-value (perm_pvalue); Chromosome location (CHR); HG19 Map position (MAPINFO); and RefSeq gene name (UCSC_RefGene_Name). Click here for file

Additional file 3: Table S2 Summary of the mean differences in methylation between FXS and control individuals. Column headers include: Illumina identification name (cpgids); Mean raw beta-values for fragile X patients (Mean FXS); Mean raw beta-values for control individuals (Mean controls); Chromosome location (CHR); HG19 Map position (MAPINFO); and RefSeq gene name (UCSC_RefGene_Name). Click here for file

Additional file 4: Figure S1 Sample age distribution. The frequency (y-axis) of FXS (orange) and control (blue) individuals at each age (x-axis), with mean ages denoted by the vertical dashed lines. Click here for file

Additional file 5: Figure S2 Methylation levels and significance of non-*FMR1* FXS-associated loci. Top two panels show the methylation levels (y-axis) in FXS (orange squares) and control (blue crosses) individuals at several loci near the two significant non-*FMR1* FXS-associated loci. Bottom two panels depict the P-values (−log(*P*-value); y-axis) for each probe generated by the mixed-effect linear model. Red line indicates the Bonferroni cutoff of 0.05. Only one probe from each region shown is significant at P <0.05. Position of probes (x-axis) in all four panels is relative to HG-19 coordinates for chromosomes 19 and 6, *KLK15* and *MICA*, respectively. Click here for file

Additional file 6: Table S3 Summary of the linear mixed-effects regression model using the data set filtered for all CGG trinucleotide repeats containing at least eight consecutive repeats (N = 136 tracts; N = 452 probes). Column headers include: Illumina identification name (cpgids); Bonferroni-corrected P-values (bonferroni); False discovery rate P-values (fdr); Uncorrected P-value (raw_pvalue); Permutation P-value (perm_pvalue); Chromosome location (CHR); HG19 Map position (MAPINFO); and RefSeq gene name (UCSC_RefGene_Name). Click here for file

Additional file 7: Figure S4 Methylation levels of *ZFHX3*. All panels show the methylation levels (y-axis) in FXS (orange squares) and control (blue crosses) individuals at loci annotated to *ZFHX3*. Top panel (All CpG probes) shows the methylation levels of all the probes annotated to *ZFHX3*. Middle panel (Probes in a CGG repeat) displays only those *ZFHX3* probes (4) that reside in a CGG trinucleotide repeat containing at least eight consecutive repeats. The bottom panel (Significant CGG repeat probes) shows the two *ZFHX3*-annotated probes that are significantly different (Bonferroni <0.05) between FXS and control individuals. Position of probes (x-axis) in all three panels is relative to HG-19 coordinates for chromosome 16. Click here for file

Additional file 8: Table S4 Summary of the linear mixed-effects regression model using the iPS cell data set filtered loci that satisfied an FDR <0.05 in the peripheral blood analysis (N = 1183 probes). Column headers include: Illumina identification name (cpgids); Bonferroni-corrected P-values (bonferroni); False discovery rate P-values (fdr); Uncorrected P-value (raw_pvalue); Chromosome location (CHR); HG19 Map position (MAPINFO); and RefSeq gene name (UCSC_RefGene_Name). Click here for file
