# Supplementary figures and images for: Genome-wide analysis validates aberrant methylation in fragile X syndrome is specific to the FMR1 locus
Source: BMC Med Genet. 2013 Jan 29;14:18. doi: 10.1186/1471-2350-14-18 (PMC3599197; doi:10.1186/1471-2350-14-18)

## Permuted vs. Asymptotic p-values

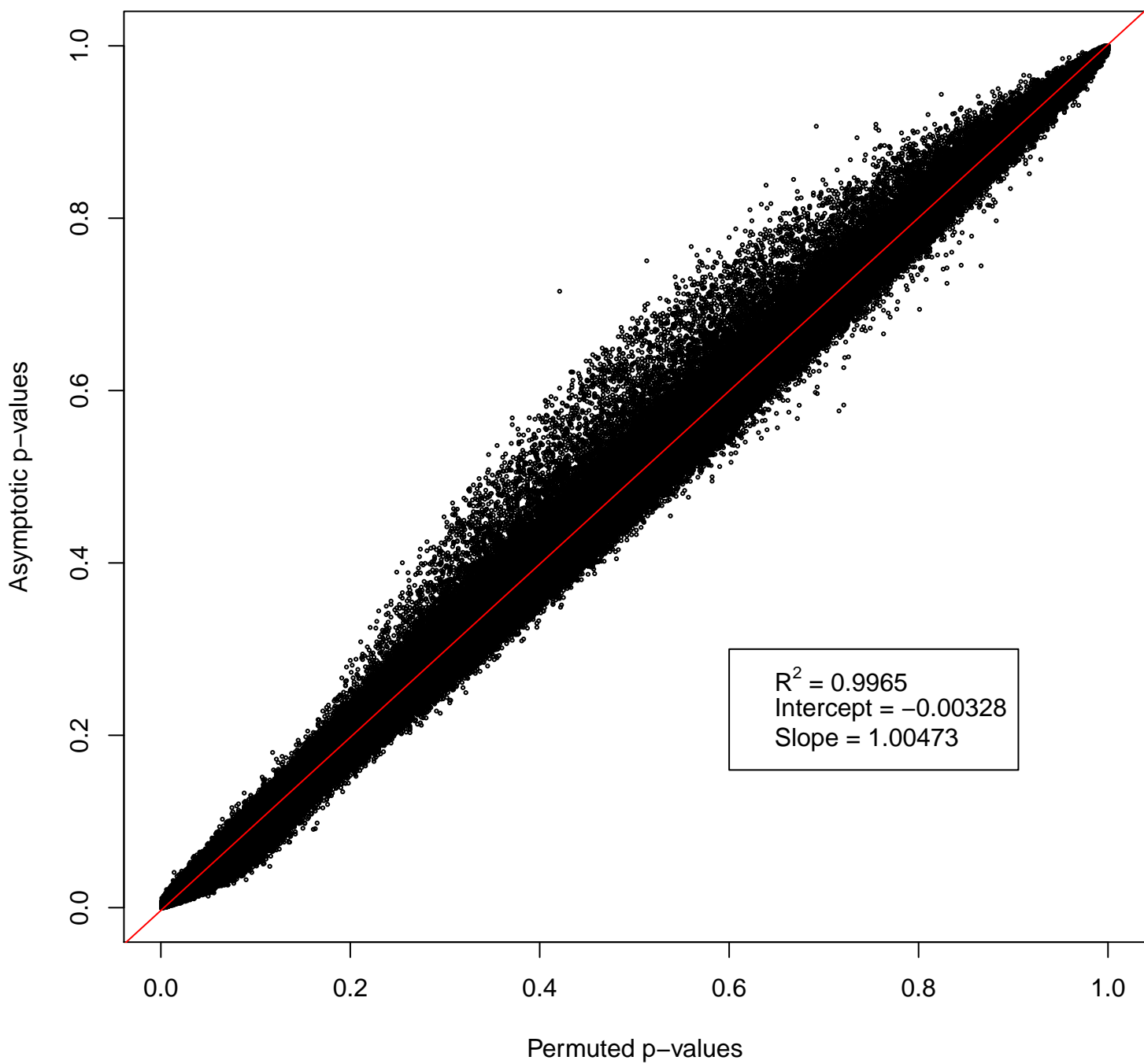

Supplement: Additional file 1: Figure S3 — Permutation analysis of FXS-associated loci. Scatterplot of permuted (1000 permutations) FXS-associated P-values (x-axis) compared to asymptotic P-values (y-axis) calculated using the linear model (Pearson R = 0.997). [file 1471-2350-14-18-S1.pdf]

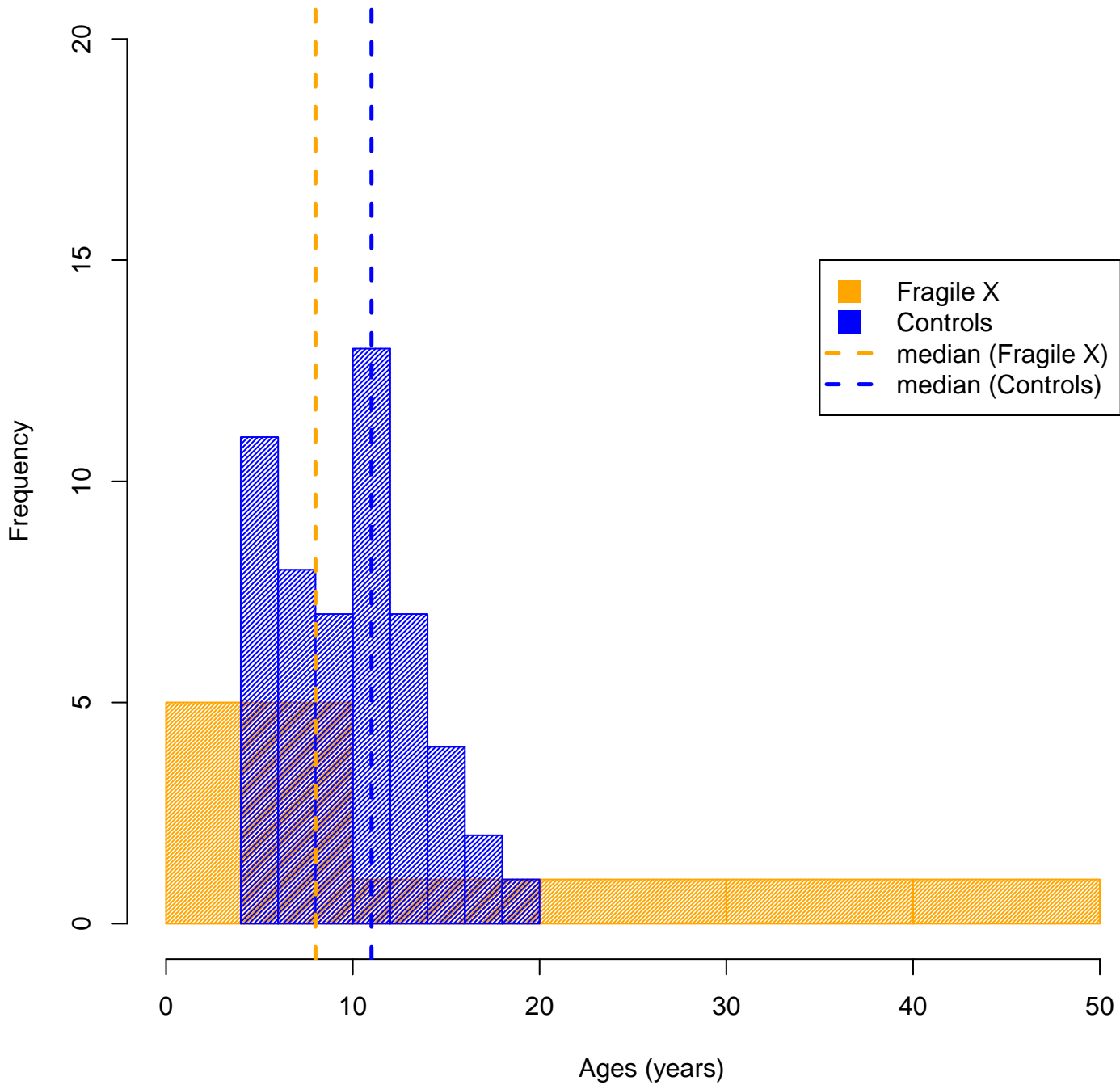

Supplement: Additional file 4: Figure S1 — Sample age distribution. The frequency (y-axis) of FXS (orange) and control (blue) individuals at each age (x-axis), with mean ages denoted by the vertical dashed lines. [file 1471-2350-14-18-S4.pdf]

KLK15

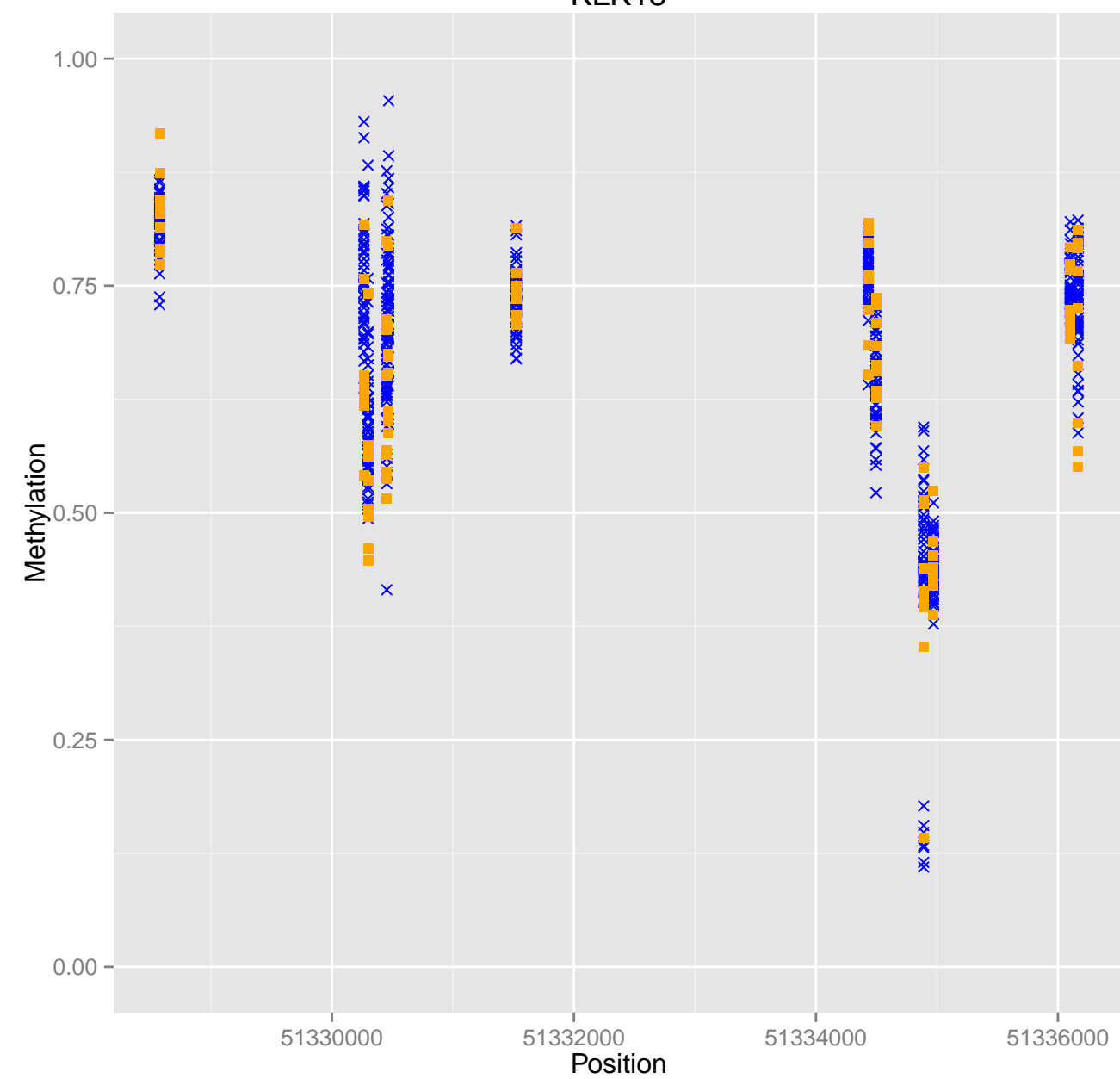

MICA

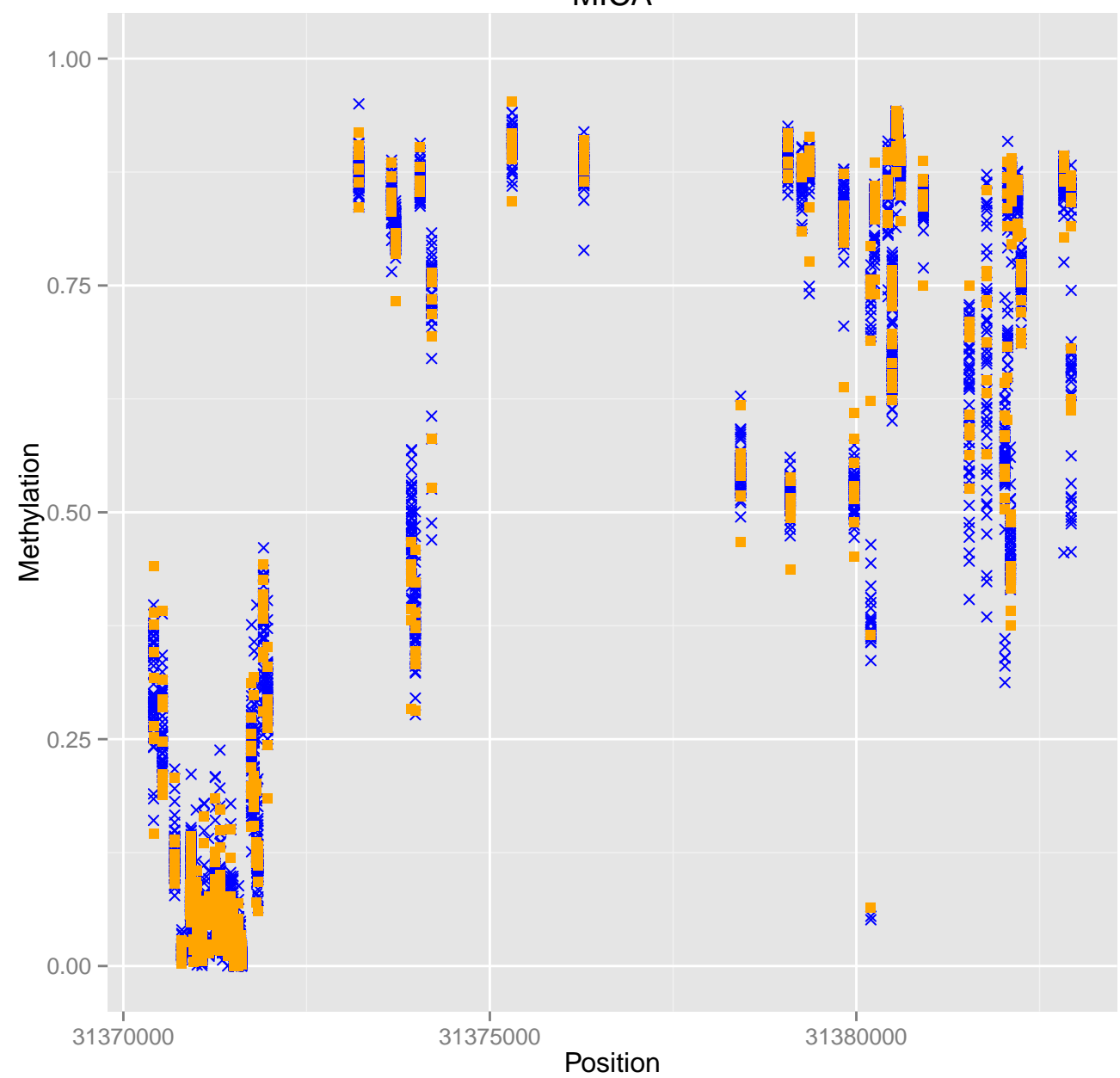

KLK15

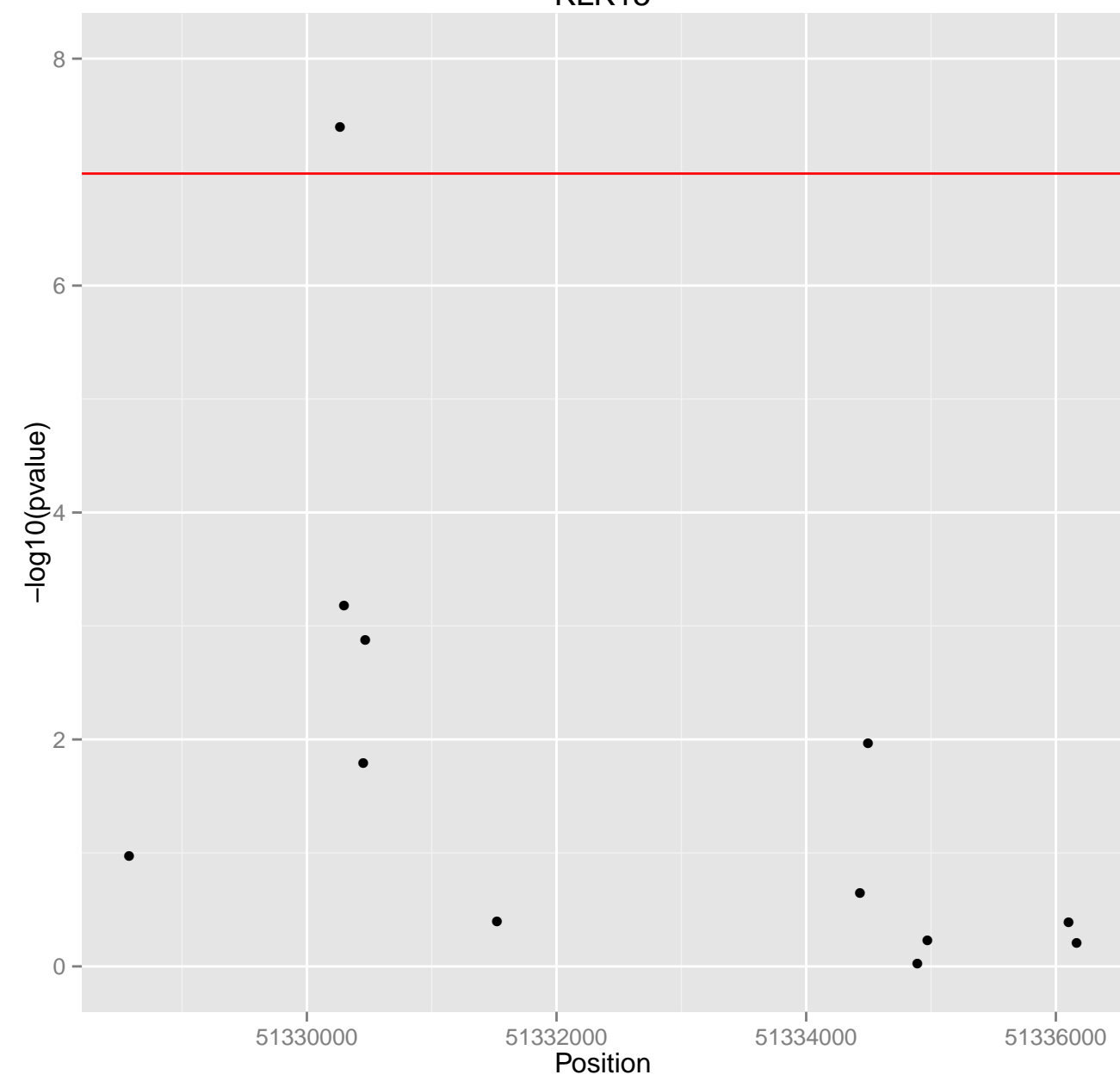

MICA

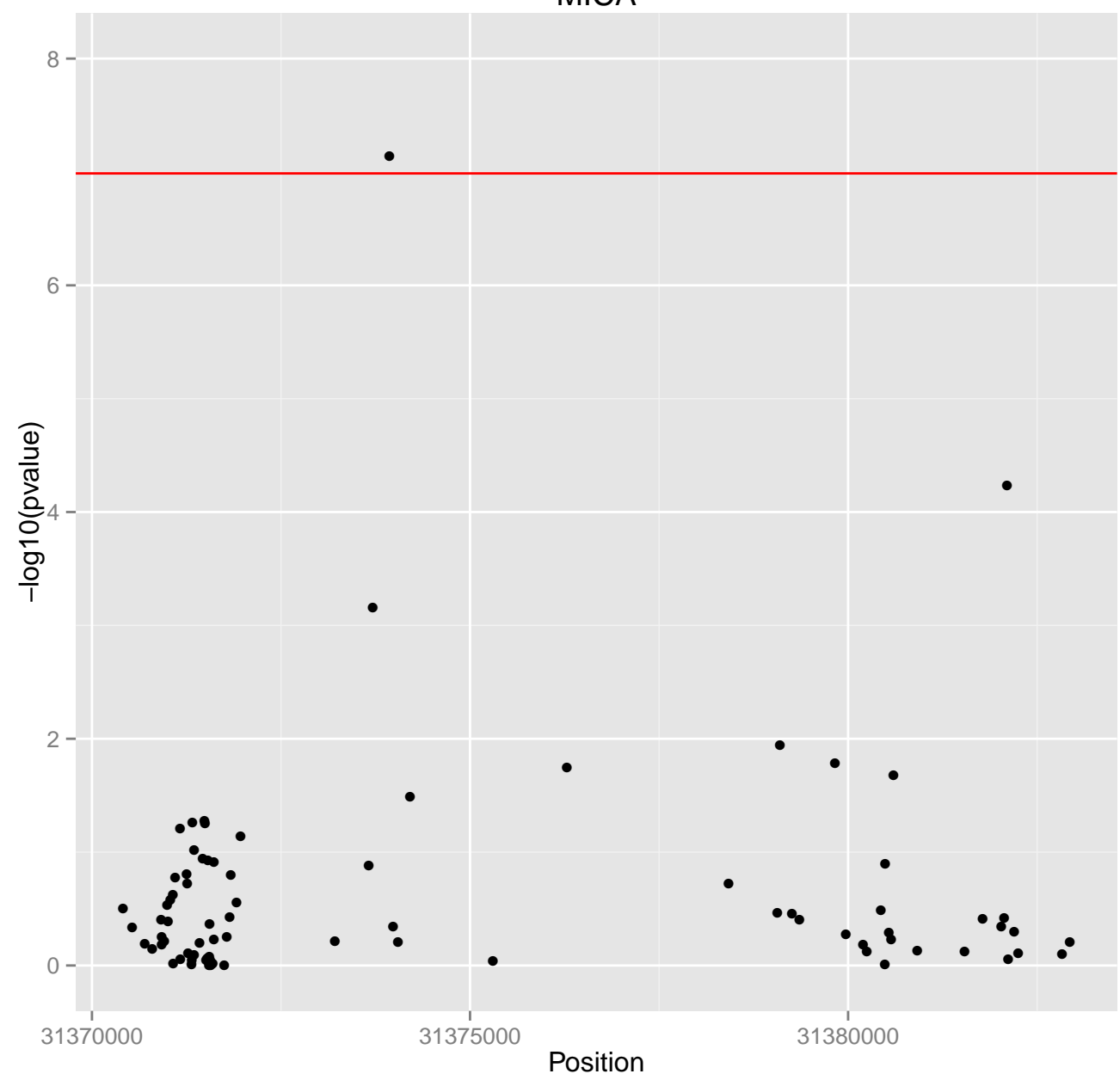

Supplement: Additional file 5: Figure S2 — Methylation levels and significance of non-FMR1 FXS-associated loci. Top two panels show the methylation levels (y-axis) in FXS (orange squares) and control (blue crosses) individuals at several loci near the two significant non-FMR1 FXS-associated loci. Bottom two panels depict the P-values (−log(P-value); y-axis) for each probe generated by the mixed-effect linear model. Red line indicates the Bonferroni cutoff of 0.05. Only one probe from each region shown is significant at P <0.05. Position of probes (x-axis) in all four panels is relative to HG-19 coordinates for chromosomes 19 and 6, KLK15 and MICA, respectively. [file 1471-2350-14-18-S5.pdf]

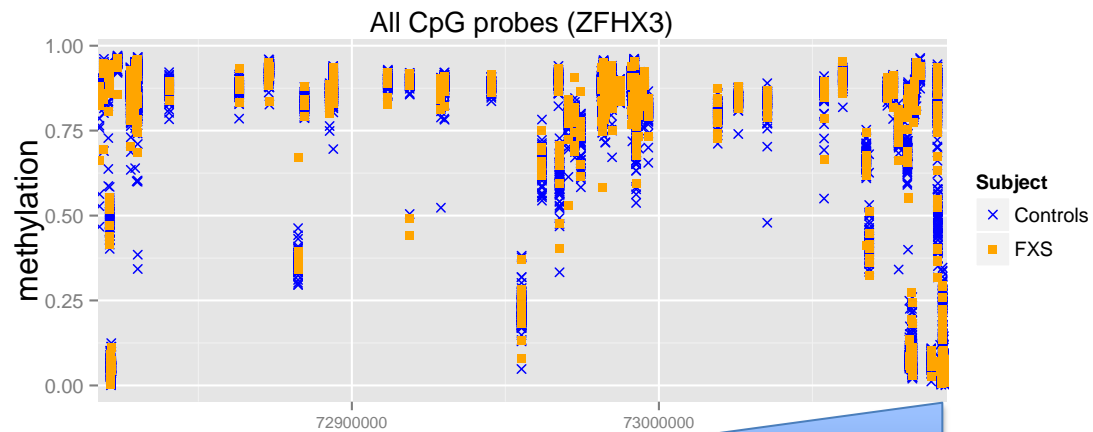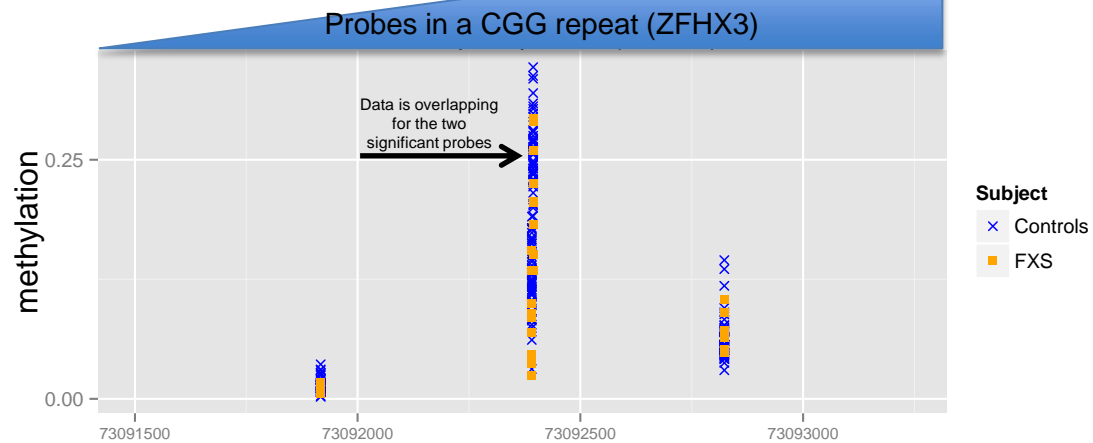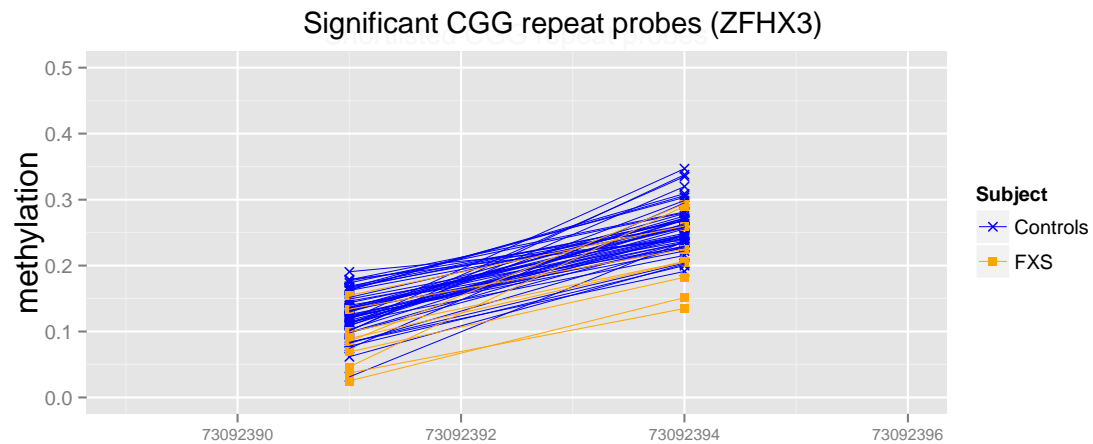

Supplement: Additional file 7: Figure S4 — Methylation levels of ZFHX3. All panels show the methylation levels (y-axis) in FXS (orange squares) and control (blue crosses) individuals at loci annotated to ZFHX3. Top panel (All CpG probes) shows the methylation levels of all the probes annotated to ZFHX3. Middle panel (Probes in a CGG repeat) displays only those ZFHX3 probes (4) that reside in a CGG trinucleotide repeat containing at least eight consecutive repeats. The bottom panel (Significant CGG repeat probes) shows the two ZFHX3-annotated probes that are significantly different (Bonferroni <0.05) between FXS and control individuals. Position of probes (x-axis) in all three panels is relative to HG-19 coordinates for chromosome 16. [file 1471-2350-14-18-S7.pdf]
